# The effect of preventative cardiovascular therapies on coronary artery disease in people with and without type 2 diabetes: a propensity-matched score study

**DOI:** 10.1186/s12872-021-02265-2

**Published:** 2021-09-26

**Authors:** Katerina V. Kiburg, Andrew I. MacIsaac, Georgia E. McCluskey, Vijaya Sundararajan, Richard J. MacIsaac

**Affiliations:** 1 Department of Endocrinology and Diabetes, St Vincent’s Hospital Melbourne, Fitzroy, VIC Australia; 2 Department of Medicine, University of Melbourne, Fitzroy, VIC Australia; 3 St Vincent’s Institute of Medical Research, Fitzroy, VIC Australia; 4 Department of Cardiology, St Vincent’s Hospital Melbourne, Fitzroy, VIC Australia; 5 Department of Public Health, La Trobe University, Bundoora, Australia

**Keywords:** Type 2 diabetes, Coronary artery disease, Coronary angiogram, Epidemiology

## Abstract

**Background:**

Although it is known that patients with Type 2 Diabetes Mellitus (T2DM) are at an increased risk of coronary artery disease (CAD), the actual coronary artery burden of atherosclerotic disease in patients with and without T2DM in a real-world setting and its possible modification by preventative therapies has not been extensively documented.

**Methods:**

Merged coronary angiography and hospital discharge data between 2013 and 2019 were obtained for analysis and a random sub-sample of patient charts were reviewed for medication use. Propensity scores were estimated using logistic regression models and used to match patients, looking at the effect of severity of CAD over time in years in an ordinal logistic regression model. A separate propensity score was estimated and used to inverse probability weight the ordinal logistic regression looking at the effect of medication use on CAD severity in patients with and without T2DM.

**Results:**

From 3,016 patients in the coronary angiography database, 1421 with T2DM and 1421 without T2DM were matched on propensity score. T2DM patients had more extensive CAD in 2018 compared to 2013 ((adjusted odds ratio) adjOR: 2.06 95% C.I. 1.38, 2.07), but this risk appeared to be attenuated in 2019. In contrast, there was no effect of time on CAD burden in patients without diabetes. In the sub-sample of 760 patients who underwent a chart review of their medication use, there were 367 (48%) with T2DM. For patients with T2DM 69.8% reported taking statins, 64.0% RAS inhibitors and 64.0% anti-platelet drugs. This was significantly higher than patients without diabetes of whom 46.6% reported taking statins, 49.0% RAS inhibitors and 49.9% anti-platelet drugs. As in the full matched sample, patients with diabetes had more extensive CAD (adjOR: 1.32 95% CI: 1.01, 1.74). However, after adjustment for the use of RAS inhibitors, statins and anticoagulants there was no difference in extent of CAD between patients with and without diabetes (adjOR: 1.14 95% CI: 0.85, 1.53).

**Conclusions:**

Although patients with diabetes have a greater extent of CAD in comparison to those without T2DM, preventative medication use decreases this CAD burden significantly.

**Supplementary Information:**

The online version contains supplementary material available at 10.1186/s12872-021-02265-2.

## Background

Patients with diabetes are known to be at an increased risk for developing coronary artery disease (CAD) compared to patients with normoglycaemia. A number of studies over the past decades have shown that the management of cardiometabolic risk factors including hyperglycaemia, dyslipidemia, and hypertension through intensive multifactorial interventions reduce the development and progression of diabetes related macrovascular complications [1–5]. Despite this, cardiovascular (CV) disease continues to be the leading cause of death for adult patients with type 2 diabetes mellitus (T2DM) and represents a substantial economic burden to both the patient and population [6, 7]. Whilst there have been a large number of clinical studies documenting the relatively poorer CV outcomes for patients with diabetes, there have been few studies that have examined the impact of diabetes on coronary artery anatomy. In particular, the extent of CAD involvement for patients with and without diabetes and the impact of traditional CV disease preventative therapies on disease within the coronary arteries, as assessed by angiography, has not been well characterised over the past 10 years. We have therefore evaluated the severity of CAD based on coronary angiogram for patients with and without T2DM admitted to a tertiary hospital in Australia over the seven-year period between 2013 and 2019. We hypothesis that people with diabetes will have a greater burden of atherosclerosis within their coronary arteries compared to those without diabetes, even following adjustment for the use of traditional CV protective medications.

## Methods

### Setting and data sources

**Fig. 1:**
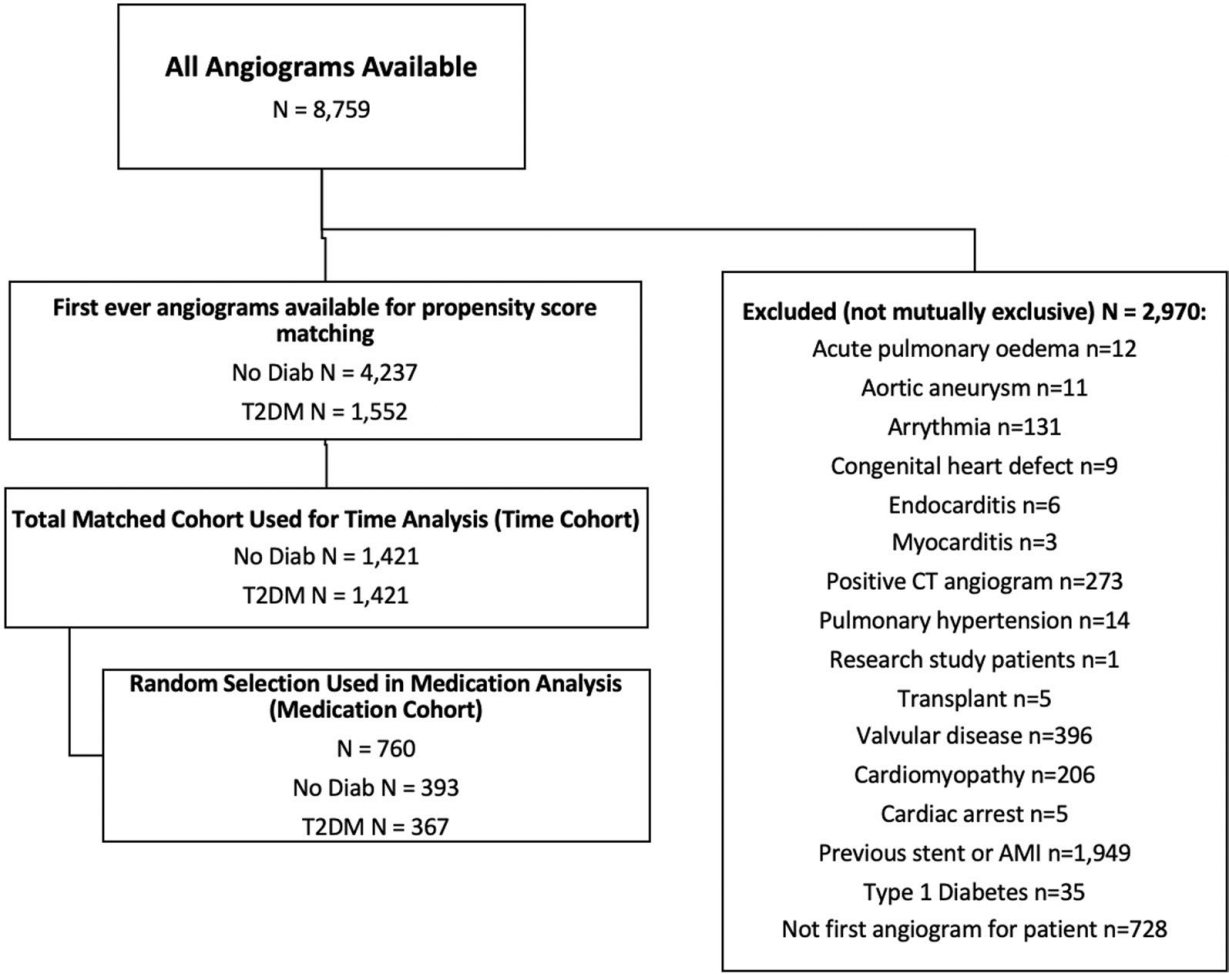
Flow chart of cohort numbers

All patient coronary angiography and hospital discharge data at a large Australian tertiary referral hospital between 2013 and 2019 were obtained and merged using the hospital record number. A random stratified sample of these patient charts were reviewed for medication use (renin-angiotensin system (RAS) inhibitors, statins, anti-platelet drugs and medications related to diabetes management), with 50% of charts selected based on T2DM diagnosis (Fig. 1). Diagnostic information is coded according to the International Statistical Classification of Diseases and Related Health Problems, Australian Modification (ICD-10-AM) [8].

### Case definition

A patient’s first known angiogram at the hospital was included. Indication for angiography was based on the original classification by treating cardiologist and included: chest pain, history of an acute coronary event, breathlessness, positive non-invasive test and other indications for angiogram which could not be directly linked with atherosclerotic disease. The sample was restricted to angiograms for the above indication and excluded angiograms performed for the following indications: acute pulmonary oedema, aortic aneurysm, arrhythmia, cardiac arrest, cardiomyopathy, congenital heart defect, endocarditis, myocarditis, positive CT coronary angiogram, previous myocardial infarction, previous stent, pulmonary hypertension, research study patients, transplant work-up or valvular disease. A diagnosis of T2DM was established using ICD-10-AM codes E11.0 to E11.9. Angiograms for patients with a diagnosis of type 1 diabetes using ICD-10-AM codes E10 were excluded.

### Main outcome

The main outcome was the extent of CAD based on the original classification by treating cardiologists: no disease, mild disease, moderate disease or severe disease. Although grading of the extent of CAD was at the discretion of the treating cardiologist, patients with mild disease had minor coronary disease without any evidence of significant vessel stenosis, those with moderate disease had at least one vessel with greater than 50% stenosis, and those with severe disease had two or three vessels with greater than 50% stenosis.

### Statistical analysis

To reduce potential confounding factors in the sample, we used the propensity score in two ways: to match and to inverse probability weight, further detail Additional file 2. The propensity scores were estimated using logistic regression models and included baseline patient characteristics (age, gender, country of birth (Australian y/n), English spoken (y/n), indication for angiogram, year of coronary angiogram, Socio-Economic Indexes for Areas (SEIFA) index (a measure of relative socio-economic disadvantage, Additional file 2) and Elixhauser comorbidities (a measure of patients disease burden using hospital administrative data) [9]. Standardised differences were calculated for each baseline patient characteristic variable, with all standardised differences being < 0.1 [10]. One propensity score was developed to identify a 1:1 matched sample of patients with and without T2DM in the time analysis with a prespecified caliper of 0.01 (the time cohort) and a second propensity score was developed to inverse probability weight the sub-sample which underwent chart review for their medication use (the medication cohort, see Fig. 1).

Continuous variables are presented as median (interquartile range) and categorical variables as total number and percentage (%). Patient characteristics by diabetes status were compared using Wilcoxon rank-sum test for continuous variables and Pearson’s $\chi^{2}$ for categorical variables.

An ordinal logistic regression model stratified by T2DM status was fitted to estimate effect of T2DM on the severity of CAD (none, mild, moderate or severe) over time in years in the matched sample, the time cohort. A separate ordinal logistic regression model was fitted to estimate the average treatment effect among the treated on the random sample of patients who had their charts reviewed for medication use, the medication cohort [11]. All analyses were conducted using STATA version 16.1 (StataCorp, College Station, TX).

## Results

Baseline patient characteristics of the study population are shown in Additional file 1: Table S1. Of first angiograms 1572 (27.2%) had T2DM. Patients with T2DM were significantly older, less likely to be born in Australia, less likely to speak English and had greater numbers of co-morbid conditions than those without T2DM. There were no significant differences in the indication for coronary angiography in patients with and without T2DM: chest pain, 22% versus 22%; history of Acute Coronary Event (ACS), 9% versus 10%; breathlessness, 8% versus 9%; positive non-invasive test, 36% versus 33% and other, 26% versus 27%, respectively.

### Time cohort

Following propensity score matching, there were 1421 matched pairs of patients (Table 1). The median age of patients was 67.3 years (IQR 59.0, 74.7) and 63.2% (1796) were male. As expected, in the matched cohort there were no significant differences for any of the covariates between patients with and without diabetes.

**Table 1:** Patient demographics for the time cohort

Characteristics	No diabetes (n = 1421)	T2DM (n = 1421)	Standardised difference (%)	*P* value
Age, median (IQR)	67.6 (58.8, 75.5)	67.0 (59.1, 73.7)	1.9	0.22
Gender (Male)	921 (64.8%)	875 (61.6%)	6.7	0.07
*Country of birth*				
Non-Australian	435 (30.6%)	432 (30.4%)	0.5	0.80
Australian	933 (65.7%)	929 (65.4%)	0.6	
Unknown	53 (3.7%)	60 (4.2%)	2.5	
*Language spoken*				
English	1253 (88.2%)	1253 (88.2%)	0.0	0.93
Not English	121 (8.5%)	124 (8.7%)	0.8	
Unknown	47 (3.3%)	44 (3.1%)	1.2	
SEIFA Index, median (IQR)	6.0 (4.0, 7.0)	6.0 (4.0, 7.0)	0.7	0.57
*Stage of CKD*				
No known CKD	1343 (94.5%)	1333 (93.8%)	3.0	0.96
CKD stage 2	6 (0.4%)	5 (0.4%)	1.1	
CKD stage 3	41 (2.9%)	45 (3.2%)	1.6	
CKD stage 4	15 (1.1%)	18 (1.3%)	2.0	
CKD stage 5	12 (0.8%)	15 (1.1%)	2.2	
CKD stage unknown	4 (0.3%)	5 (0.4%)	1.3	
*Indication for angiogram*				
Chest pain	317 (22.3%)	315 (22.2%)	0.2	1.00
History of acute coronary event	120 (8.4%)	121 (8.5%)		
Breathlessness	115 (8.1%)	119 (8.4%)		
Positive non-invasive test	504 (35.5%)	506 (35.6%)		
Other	365 (25.7%)	360 (25.3%)		
*Year of angiogram*				
2013	141 (9.9%)	147 (10.3%)	2.0	0.95
2014	210 (14.8%)	207 (14.6%)		
2015	208 (14.6%)	206 (14.5%)		
2016	209 (14.7%)	220 (15.5%)		
2017	229 (16.1%)	242 (17.0%)		
2018	226 (15.9%)	209 (14.7%)		
2019	198 (13.9%)	190 (13.4%)		
Valvular disease	140 (9.9%)	145 (10.2%)	1.2	0.75
Peripheral vascular disorders	30 (2.1%)	34 (2.4%)	1.9	0.61
Congestive heart failure	133 (9.4%)	135 (9.5%)	0.5	0.90
Cardiac arrhythmia	103 (7.2%)	107 (7.5%)	1.1	0.77
Hypertension	201 (14.1%)	190 (13.4%)	2.2	0.55

Other Elixhauser comorbidities matched included; pulmonary circulation disorders, other neurological disorders, chronic obstructive pulmonary disease, renal failure, liver disease, metastatic cancer, solid tumour without metastasis, rheumatoid arthritis/collagen vascular disease, coagulopathy, obesity, weight loss, fluid and electrolyte disorders, blood loss anaemia, deficiency anaemia, alcohol abuse and depression

**Fig. 2:**
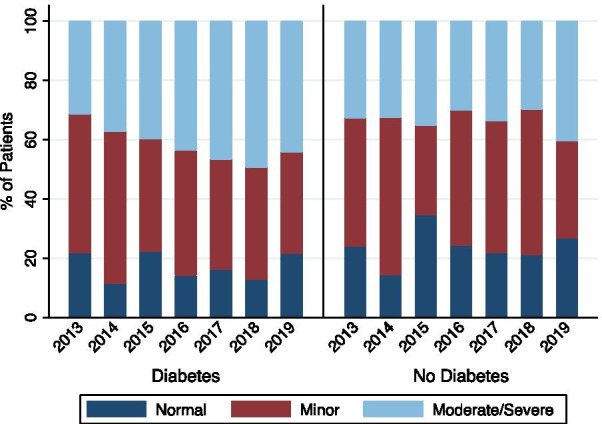
Extent of CAD for patients with and without diabetes of time cohort

Additional file 1: Table S2 lists the extent of CAD over time. Figure 2 shows the change in extent of CAD in patients both with and without T2DM over the period of the study. In the ordinal logistic regression, an increasing risk of more extensive CAD was observed between 2013 and 2018 (OR: 2.06 95% C.I. 1.38, 3.09) (Table 2) for patients with diabetes. However, this risk appeared to be attenuated in 2019 with no significant change in severity of CAD in 2013 compared with 2019 (OR: 1.37 95% C.I. 0.91, 2.07) (Table 2). No consistent significant effect of time on CAD burden in patients without diabetes was found (Table 2).

**Table 2:** Ordinal logistic regression model for extent of CAD in the time cohort

Extent of CAD	No diabetes		T2DM	
	Odds ratio (95% CI)	*P* value	Odds ratio (95% CI)	*P* value
Age	1.05 (1.05, 1.06)	< 0.001	1.04 (1.03, 1.05)	< 0.001
Gender (male)	2.44 (1.97, 3.01)	< 0.001	2.21 (1.80, 2.72)	< 0.001
*Study year*				
2013	Reference		Reference	
2014	1.09 (0.73, 1.63)	0.66	1.49 (1.00, 2.21)	0.05
2015	0.85 (0.56, 1.28)	0.43	1.30 (0.87, 1.94)	0.20
2016	0.95 (0.63, 1.42)	0.79	1.60 (1.08, 2.38)	0.02
2017	1.07 (0.72, 1.58)	0.75	1.77 (1.20, 2.61)	< 0.001
2018	0.94 (0.63, 1.40)	0.76	2.06 (1.38, 3.09)	< 0.001
2019	1.19 (0.78, 1.80)	0.42	1.37 (0.91, 2.07)	0.13

### Medication cohort

Of the 760 patients with medication details available, 367 (48.0%) also had a diagnosis of T2DM. Following propensity score weighting no significant differences between any patient covariates persisted (Table 3).

**Table 3:** Patient demographics for the medication cohort

Characteristics	No diabetes (n = 393)	T2DM (n = 367)	Standardised difference (%)	*P* value
Age, median (IQR)	63.9 (54.4, 73.0)	66.6 (59.9, 72.7)	0.5	0.95
Gender (male)	230 (58.5%)	224 (61.0%)	1.5	0.84
*Country of birth (Australian)*				
Non-Australian	92 (23.4%)	108 (29.4%)	0.7	0.85
Australian	289 (73.5%)	239 (65.1%)	0.2	
Unknown	12 (3.1%)	20 (5.4%)	2.1	
SEIFA Index, median (IQR)	6.0 (4.0, 7.0)	6.0 (4.0, 7.0)	1.2	0.88
*Stage of CKD*				
No known CKD	381 (96.9%)	337 (91.8%)	0.3	0.97
CKD stage 2	1 (0.3%)	2 (0.5%)	4.9	
CKD stage 3	5 (1.3%)	21 (5.7%)	4.0	
CKD stage 4	2 (0.5%)	3 (0.8%)	1.5	
CKD stage 5	4 (1.0%)	4 (1.1%)	1.0	
*Indication for angiogram*				
Chest pain	93 (23.7%)	91 (24.8%)	0.6	0.94
History of acute coronary event	45 (11.5%)	42 (11.4%)		
Breathlessness	42 (10.7%)	32 (8.7%)		
Positive non-invasive test	144 (36.6%)	146 (39.8%)		
Other	69 (17.6%)	56 (15.3%)		
*Year of angiogram*				
2013	57 (14.5%)	37 (10.1%)	1.6	0.84
2014	46 (11.7%)	50 (13.6%)		
2015	53 (13.5%)	56 (15.3%)		
2016	54 (13.7%)	56 (15.3%)		
2017	60 (15.3%)	55 (15.0%)		
2018	60 (15.3%)	51 (13.9%)		
2019	63 (16.0%)	62 (16.9%)		
Valvular disease	39 (9.9%)	25 (6.8%)	2.6	0.77
Peripheral vascular disorders	14 (3.6%)	5 (1.4%)	4.3	0.71
Congestive heart failure	32 (8.1%)	23 (6.3%)	< 0.01	1.00
Cardiac arrhythmia	31 (7.9%)	27 (7.4%)	0.9	0.91
Hypertension	41 (10.4%)	53 (14.4%)	1.4	0.86

Other Elixhauser comorbidities matched included; pulmonary circulation disorders, chronic obstructive pulmonary disease, renal failure, liver disease, solid tumour without metastasis, coagulopathy, weight loss, fluid and electrolyte disorders, deficiency anaemia and alcohol abuse

When diabetes medications use were examined, metformin was the most frequently reported (66.5%), followed by other medications, which included thiazolidinediones and sulfonylureas (36.8%) and insulin (23.4%). Very few patients reported taking medications belonging to the relatively newer classes of glucoses lowering medications, the sodium-glucose transport-2 (SGLT-2) inhibitors (4.9%) or glucagon-like peptide-1 (GLP-1) receptor agonists (3.3%) (Additional file 1: Table S3). Patients with T2DM were more likely to take all of the traditional CV disease preventative therapies than patients without T2DM (Additional file 1: Table S3). The greatest difference in medication use between patients with and without T2DM was seen for the use of statins, 69.8% compared to 46.6% (*p* < 0.001), respectively. Patients with T2DM were also more likely to be prescribed RAS inhibitors compared to those without T2DM, 64.0% versus 49.0% (*p* < 0.001), respectively. There was also a small but significant difference in the rates of anti-platelet drug use in patients with and without T2DM, 64.0% versus to 49.9% (*p* = 0.05), respectively.

Of patients with T2DM, 78.2% (n = 287) had evidence of angiographic CAD compared with 70.2% (n = 276) of patients without T2DM, *p* < 0.01 (Additional file 1: Table S4). In the unweighted ordinal logistic regression model patients with T2DM were significantly more likely to have extensive CAD than patients without T2DM (OR: 1.32 95% CI: 1.01, 1.74). Both increasing age (OR: 1.06 95% CI: 1.04, 1.07) and being male (OR: 1.80 95% CI: 1.36, 2.38) were associated with more extensive CAD in patients with information about medication use (Table 4). Following propensity score weighting and adjustment for the use of statins, anti-platelet drugs and RAS inhibitors, no significant difference in extent of CAD was seen between patients with and without T2DM (OR: 1.14 95% CI: 0.85, 1.53). In the multivariate regression model both age and being male were still associated with more extensive CAD. The use of anti-platelet drugs was also associated with more extensive CAD (OR: 2.64 95% CI: 1.93, 3.61) (Table 4).

**Table 4:** Extent of CAD for the medication cohort

	Odds Ratio (95% CI)	*P* value
*Unadjusted ordinal logistic regression model*		
Diabetes	1.32 (1.01, 1.74)	0.04
Age	1.06 (1.04, 1.07)	< 0.001
Gender (male)	1.80 (1.36, 2.38)	< 0.001
*Adjusted ordinal logistic regression model following propensity score matching*		
Diabetes	1.14 (0.85, 1.53)	0.39
Age	1.05 (1.03, 1.06)	< 0.001
Gender (male)	1.76 (1.30, 2.39)	< 0.001
Statin (yes)	1.15 (0.84, 1.59)	0.38
Antiplatelet drug (yes)	2.64 (1.93, 3.61)	< 0.001
RAS inhibitor (yes)	1.37 (1.01, 1.86)	0.04

## Discussion

We found that T2DM patients have a greater burden of CAD as assessed by angiography compared to those without diabetes. Interestingly we also found that T2DM patients had more extensive CAD as assessed by angiograms performed in 2018 compared to 2013, but that possibly this trend was attenuated in 2019. The above findings need to be put into the context of a possible change in the indication or thresholds for performing coronary angiograms in people with T2DM over the observational period of the study. However, possibly the most important finding from our study is that despite a greater use of CV protective medications by patients with T2DM, the extent of disease within the coronary arteries, as assessed by angiography, was still greater in patients with T2DM compared to those without T2DM. However, after adjustment for medication use, we found that there was no longer any difference in the extent of CAD between patients with and without T2DM. These findings highlight the exaggerated risk that patients with T2DM are still at for developing CAD. However, they also suggest that with the greater application of traditional CV disease preventative therapies, it may be possible to reduce the extent of the CAD burden in patients with diabetes to that seen in patients without T2DM.

Of the 367 patients with T2DM and medication details available, 78% (n = 287) had evidence of angiographic disease. This rate was less in patients without T2DM, of which 70% (n = 276) had evidence of angiographic CAD. In our unadjusted ordinal logistic regression model, prior to accounting for the use of preventative medications, patients with T2DM were significantly more likely (by 32%) to have more extensive CAD than patients without T2DM (OR: 1.32 95% CI: 1.01, 1.74). Following adjustment for the use of statins, RAS inhibitors and anti-platelet drugs, this significant difference in extent of CAD no longer persisted between patients with and without T2DM (OR: 1.14 95% CI: 0.85, 1.53).

When comparing the use of traditional CV disease preventative therapies between patients with and without T2DM; statins, RAS inhibitors and anti-platelet drugs were significantly more likely to be taken by patients with T2DM. The increase in medication use for patients with T2DM compared to those without T2DM was 23% for statins, 14% for RAS inhibiting agents and 7% for anti-platelet agents. All of these differences for the uptake of CV preventative medication in patients with T2DM compared to those without T2DM were statistically significant. Whilst these results are encouraging, our results also emphasise that more aggressive uptake and use of CV protective medications, possibly earlier after a diagnosis of T2DM is made, is still required to reduce the gap between the heightened risk for more severe CAD in patients with T2DM compared to those without T2DM. Unfortunately, we lacked information on achieved lipid or blood pressure measurements to gauge whether targeting the above medications to achieve certain biochemical or clinical parameters influenced the results we report.

It is well established that risk factor modification through the optimisation of glycated haemoglobin levels and blood pressure, treatment of microalbuminuria, dietary intervention, exercise and smoking cessation are able to reduce the risk in the development and progression of vascular complications [4, 12]. Our findings appear to be broadly consistent with those of a recent large epidemiological study which has also suggested that it is possible for patients with T2DM to eliminate their excess risk for acute myocardial infarction compared to the general population if they can achieve five risk-factor variables (glycated haemoglobin, low-density lipoprotein cholesterol, systolic blood pressure, albuminuria and smoking status) within target ranges/recommendations [5].

A number of trials, both pharmacological and epidemiological, have suggested that the use of statins and blood pressure (BP) lowering medications are most likely responsible for the change in risk for CV events that had been observed in recent times [13–17]. The publication of these large influential studies in the 1990’s and early 2000’s and the further confirmation of the CV benefits of the above approach in later studies has led to a drastic increase in the uptake of statins and anti-hypertensive agents, in particular RAS inhibitors, for both high risk vascular patients with and without T2DM [18–20]. However, over time, some concern about side-effects has been reported regarding the use of statins [21]. If patients had been ceasing statins due to this concern, we would have expected to see the effect equally across both patient groups with and without diabetes. Given the reported side effects associated with statins, the wider use of newer approaches to treat dyslipidaemia such as the PCSK-9 inhibitors and possibly, ethyl icosapent, may result in a further decrease in the CAD burden in both patient groups with and without diabetes [22].

Targets for optimising BP lowering therapy in patients with and without diabetes still remain to be optimally defined and indeed, there has been a relaxing of BP targets for patients with diabetes over the last 10 years [23]. However, RAS inhibiting agents are often prescribed for the cardio-renal effects in addition to their use as BP lowering agents. Unfortunately, the design of our study did not allow us to follow any temporal trends in number of patients with and without T2DM taking these agents. Furthermore, the value of aspirin therapy as a primary CV preventative agent, when offset by potential adverse events, has also been called into question in recent times. Whether this issue has differentially affected the use of aspirin therapy in patients with and without diabetes remains to be examined.

Our analysis of patients admitted for their first coronary angiogram at a single tertiary hospital shows that the extent of CAD being detected on coronary angiogram was greater over time in patients with T2DM. This was in contrast to patients without T2DM where no significant effect on time on CAD extent was demonstrated. These findings appear to be contrary to reports of reduced rates for myocardial infarctions over recent years for patients with and without diabetes, with relative rate reductions appearing to be being larger in patients with T2DM [24]. It is therefore important to emphasise that the results we report are not based on clinical events or outcomes but rather on findings from a coronary angiogram. It is possible that the initial increase in severity of CAD burden seen in our study for patients with T2DM may represent improved CAD screening over recent years rather than suboptimal application of preventative therapies to slow disease progression within the coronary arteries. A lower threshold for performing a coronary angiogram may also exist for patients with T2DM given the appreciated higher risk of CAD a diagnosis of T2DM confers and a higher appreciation of asymptomatic CAD in patients with T2DM [25]. Possibly, wider use of medications such as SGLT-2 inhibitors and GLP-1 receptor agonists which have been shown to have CV protective effects will result in improvements in CAD burden following 2019 for patients with diabetes, however a longer duration of follow-up will be required to confirm whether the attenuation of severity in CAD is a true finding [26–29].

There were a number of limitations with this study. As we utilised hospital discharge coding data, information was not available on metabolic and blood pressure control or duration of diabetes. Therefore, we are unable to account for the effect that these preventative medications may have had on patient’s risk profile. This is a single-site study, reporting on the extent of CAD in patients attending a public tertiary referral and university teaching hospital with a state-wide catchment. However, the outcome of extent of CAD may be influenced by individual differences in hospital characteristics. There may also be a small number of patients that may have incorrectly been classified as either having had or not had T2DM [30]. However, the prevalence of diabetes that we found in our study of 28% is consistent with other local studies [31]. Another limitation to consider is that this study relied on individual cardiologists reporting on the indication for angiogram and the extent of CAD without a prospective set of criteria for grading the extent of CAD; however, this is balanced by the large number of experienced cardiologists reporting results. As patients with and without T2DM were assessed in an identical fashion, it is unlikely that the extent of CAD would have been recorded disproportionately for the two groups of patients. We report on the first recorded angiogram at our hospital, it is therefore possible that a patient may have received an angiogram or coronary invasive procedure previously at a different hospital. In this case, these patients may be considered to be high risk to warrant a second angiogram and this may occur more frequently in patients with T2DM. If this was the case, these patients would almost certainly be classified as high risk vascular patients and hence been more likely to be further investigated with another angiogram at our hospital. It is presumed that the above scenario occurred more frequently in patients with diabetes given the known exaggerated risk for CAD that this group is at.

These limitations are counterbalanced by the strengths of the study which include a large number of coronary angiograms from both patients with and without diabetes over a seven year period. As it was an observational study, propensity scores were used in the regressions in order to include as many coronary angiogram results from patients with diabetes as possible allowing for a representative analysis.

## Conclusion

Our findings show that the extent of disease within the coronary arteries of patients with T2DM still remains more severe compared to those without T2DM. However, it is possible that this excess burden of disease can be attenuated to the levels seen in patients without T2DM by the more aggressive uptake of traditional CV protective medications. These results support those of a recent large epidemiological study that suggested that aggressive risk factor modification in patients with T2DM can eliminate the excess risk for myocardial infarction compared with that of the general population [5].

## Supplementary Information

**Additional file 1**: Supplementary Tables and Figures.

**Additional file 2**: Supplementary Methods.

## Data Availability

The data sets analysed during the current study are not publicly available because of information governance restrictions.

## Abbreviations

CAD: Coronary artery disease
adjOR: Adjusted odds ratios
CV: Cardiovascular
T2DM: Type 2 diabetes mellitus
RAS-inhibitors: Renin-angiotensin system-inhibitors
SEIFA: Socio-Economic Indexes for Areas
ACS: Acute Coronary Event
SGLT-2: Sodium-glucose transport-2
GLP-1: Glucagon-like peptide-1
BP: Blood pressure
ICD-10-AM: International statistical classification of diseases and related health problems, Australian modification
