# Supplementary material for: The effect of preventative cardiovascular therapies on coronary artery disease in people with and without type 2 diabetes: a propensity-matched score study
Source: BMC Cardiovasc Disord. 2021 Sep 26;21:463. doi: 10.1186/s12872-021-02265-2 (PMC8474817; doi:10.1186/s12872-021-02265-2)
Supplement: Supplementary file 1 — Additional file 1: Supplementary Tables and Figures. [file 12872_2021_2265_MOESM1_ESM.docx]

**Supplementary Tables**

**Supp Table 1: Baseline Patient Demographics**

| **Characteristics** | **No Diabetes**  **(n=4,212)** | **T2DM**  **(n=1,577)** | **p-value** |
| --- | --- | --- | --- |
| **Age, median (IQR)** | 65.0 (55.8-72.9) | 67.4 (59.7-74.1) | <0.001 |
| **Gender (Male)** | 2,568 (61.0%) | 986 (62.5%) | 0.28 |
| **Country of Birth** |  |  |  |
| Non-Australian | 957 (22.7%) | 504 (32.0%) | <0.001 |
| Australian | 3,087 (73.3%) | 992 (62.9%) |  |
| Unknown | 168 (4.0%) | 81 (5.1%) |  |
| **Language Spoken** |  |  |  |
| English | 3,854 (91.5%) | 156 (9.9%) | <0.001 |
| Not English | 192 (4.6%) | 1,329 (84.3%) |  |
| Unknown | 166 (3.9%) | 92 (5.8%) |  |
| **SEIFA* Index, median (IQR)** | 6.0 (4.0-7.0) | 6.0 (4.0-7.0) | 0.43 |
| **Stage of Chronic kidney disease (CKD)** |  |  |  |
| No known CKD | 4,092 (97.2%) | 1,422 (90.2%) | <0.001 |
| CKD stage 1 | 0 (0.0%) | 1 (0.1%) |  |
| CKD stage 2 | 8 (0.2%) | 8 (0.5%) |  |
| CKD stage 3 | 62 (1.5%) | 91 (5.8%) |  |
| CKD stage 4 | 23 (0.5%) | 22 (1.4%) |  |
| CKD stage 5 | 20 (0.5%) | 23 (1.5%) |  |
| CKD stage unknown | 7 (0.2%) | 10 (0.6%) |  |
| **Indication for Angiogram** |  |  |  |
| Chest pain | 916 (21.7%) | 344 (21.8%) | 0.11 |
| History of Acute Coronary Event | 414 (9.8%) | 134 (8.5%) |  |
| Breathlessness | 379 (9.0%) | 128 (8.1%) |  |
| Positive Non-Invasive Test | 1,373 (32.6%) | 566 (35.9%) |  |
| Other | 1,130 (26.8%) | 405 (25.7%) |  |
| **Year of Angiogram** |  |  |  |
| 2013 | 607 (14.4%) | 160 (10.1%) | <0.001 |
| 2014 | 589 (14.0%) | 246 (15.6%) |  |
| 2015 | 591 (14.0%) | 234 (14.8%) |  |
| 2016 | 589 (14.0%) | 239 (15.2%) |  |
| 2017 | 650 (15.4%) | 274 (17.4%) |  |
| 2018 | 621 (14.7%) | 219 (13.9%) |  |
| 2019 | 565 (13.4%) | 205 (13.0%) |  |
| **Valvular disease** | 405 (9.6%) | 164 (10.4%) | 0.37 |
| **Pulmonary circulation disorders** | 45 (1.1%) | 21 (1.3%) | 0.41 |
| **Peripheral vascular disorders** | 111 (2.6%) | 36 (2.3%) | 0.51 |
| **Other neurological disorders** | 12 (0.3%) | 2 (0.1%) | 0.38 |
| **Chronic obstructive pulmonary disease** | 19 (0.5%) | 7 (0.4%) | 1.00 |
| **Hypothyroidism** | 0 (0.0%) | 1 (0.1%) | 0.27 |
| **Renal Failure** | 59 (1.4%) | 81 (5.1%) | <0.001 |
| **Liver Disease** | 53 (1.3%) | 17 (1.1%) | 0.69 |
| **Peptic ulcer disease, excluding bleeding** | 1 (0.0%) | 1 (0.1%) | 0.47 |
| **HIV/AIDS** | 1 (0.0%) | 0 (0.0%) | 1.00 |
| **Lymphoma** | 0 (0.0%) | 3 (0.2%) | 0.02 |
| **Metastatic Cancer** | 3 (0.1%) | 2 (0.1%) | 0.62 |
| **Solid tumour without metastasis** | 13 (0.3%) | 9 (0.6%) | 0.15 |
| **Rheumatoid arthritis/collagen vascular disease** | 11 (0.3%) | 4 (0.3%) | 1.00 |
| **Coagulopathy** | 17 (0.4%) | 5 (0.3%) | 0.81 |
| **Obesity** | 4 (0.1%) | 6 (0.4%) | 0.03 |
| **Weight loss** | 30 (0.7%) | 13 (0.8%) | 0.73 |
| **Fluid and electrolyte disorders** | 122 (2.9%) | 60 (3.8%) | 0.09 |
| **Blood loss anaemia** | 2 (0.0%) | 2 (0.1%) | 0.30 |
| **Deficiency anaemia** | 18 (0.4%) | 19 (1.2%) | <0.01 |
| **Alcohol abuse** | 42 (1.0%) | 6 (0.4%) | 0.02 |
| **Drug abuse** | 13 (0.3%) | 1 (0.1%) | 0.13 |
| **Psychoses** | 2 (0.0%) | 1 (0.1%) | 1.00 |
| **Depression** | 19 (0.5%) | 4 (0.3%) | 0.35 |
| **Congestive heart failure** | 393 (9.3%) | 157 (10.0%) | 0.48 |
| **Cardiac arrhythmia** | 433 (10.3%) | 123 (7.8%) | <0.01 |
| **Hypertension** | 381 (9.0%) | 240 (15.2%) | <0.001 |

*Socio-economic Indexes for Areas

**Supp Figure 1: Distribution of propensity scores before and after matching, time cohort**

**Supp Table 2: Extent of CAD for patients with and without diabetes derived from the time cohort**

|  | **No T2DM* (n=1,421)** | | | **T2DM (n=1,421)** | | |
| --- | --- | --- | --- | --- | --- | --- |
| **Study Year** | **Normal** | **Minor** | **Moderate/Severe** | **Normal** | **Minor** | **Moderate/Severe** |
| 2013 | 34 (24.1%) | 61 (43.3%) | 46 (32.6%) | 32 (21.8%) | 69 (46.9%) | 46 (31.3%) |
| 2014 | 30 (14.3%) | 112 (53.3%) | 68 (32.4%) | 24 (11.6%) | 106 (51.2%) | 77 (37.2%) |
| 2015 | 72 (34.6%) | 63 (30.3%) | 73 (35.1%) | 46 (22.3%) | 78 (37.9%) | 82 (39.8%) |
| 2016 | 51 (24.4%) | 95 (45.5%) | 63 (30.1%) | 31 (14.1%) | 93 (42.3%) | 96 (43.6%) |
| 2017 | 50 (21.8%) | 102 (44.5%) | 77 (33.6%) | 39 (16.1%) | 90 (37.2%) | 113 (46.7%) |
| 2018 | 48 (21.2%) | 111 (49.1%) | 67 (29.6%) | 27 (12.9%) | 79 (37.8%) | 103 (49.3%) |
| 2019 | 53 (26.8%) | 65 (32.8%) | 80 (40.4%) | 41 (21.6%) | 65 (34.2%) | 84 (44.2%) |
| **Total** | 338 (23.8%) | 609  (42.9%) | 474  (33.4%) | 240  (16.9%) | 580  (40.8%) | 601  (42.3%) |

*Type 2 Diabetes Mellitus

**Supp Table 3: Medication use in patients with and without diabetes for the medication cohort**

| **Medication** | **No T2DM* (n=393)** | **T2DM (n=367)** | **p-value** |
| --- | --- | --- | --- |
| **Statin (Yes)** | 183 (46.6%) | 256 (69.8%) | <0.001 |
| **Antiplatelet Drug (Yes)** | 239 (60.8%) | 248 (67.6%) | 0.05 |
| **RAS Inhibitor (Yes)** | 196 (49.9%) | 235 (64.0%) | <0.001 |
| **Metformin (Yes)** | N/A | 244 (66.5%) |  |
| **Other (Yes)** | N/A | 135 (36.8%) |  |
| **Diet Only (Yes)** | N/A | 67 (18.3%) |  |
| **Insulin (Yes)** | N/A | 86 (23.4%) |  |
| **SGLT2 Inhibitors (Yes)** | N/A | 18 (4.9%) |  |
| **GLP1 Receptor Agonists (Yes)** | N/A | 12 (3.3%) |  |

*Type 2 Diabetes Mellitus

**Figure 2: Distribution of propensity scores used for propensity weighting, medication cohort**

**Supp Table 4: Extent of CAD by diabetes status for the medication cohort**

| **Extent of CAD** | **No Diabetes (n=393)** | **T2DM (n=367)** | **p-value** |
| --- | --- | --- | --- |
| **Normal** | 117 (29.8%) | 80 (21.8%) | <0.01 |
| **Minor** | 183 (46.6%) | 168 (45.8%) |  |
| **Moderate/Severe** | 93 (23.7%) | 119 (32.4%) |  |
