# Supplementary material for: The effect of preventative cardiovascular therapies on coronary artery disease in people with and without type 2 diabetes: a propensity-matched score study
Source: BMC Cardiovasc Disord. 2021 Sep 26;21:463. doi: 10.1186/s12872-021-02265-2 (PMC8474817; doi:10.1186/s12872-021-02265-2)
Supplement: Supplementary file 2 — Additional file 2: Supplementary Methods. [file 12872_2021_2265_MOESM2_ESM.docx]

**Supplementary Methods**

Definition of Socio-Economic Indexes for Areas:

Within Australia SES is measured by the Australian Bureau of Statistics (ABS) as the Socio-Economic Indexes for Areas (SEIFA) score which is a summary of an area’s relative advantage/disadvantage ranging from 1 to 10. This index is calculated on information collected at the time of the census such as(1);

- Percentage of low-income households
- Unemployment rate
- Percentage of low-skilled occupants or individuals without qualifications
- Percentage of households without a car
- Percentage of households experiencing overcrowding
- Percentage of individuals under 70 with a disability
- Percentage of children with unemployed parents
- Percentage of people with poor English proficiency

Reference:

1. Australian Bureau of Statistics. Socio-Economic Indexes for Areas (SEIFA) 2016 Technical Paper. Australian Bureau of Statistics Canberra, Australia,; 2018.
